# Real-world use of Safinamide in motor fluctuating Parkinson’s disease patients in Italy

**DOI:** 10.1007/s10072-023-07001-6

**Published:** 2023-09-09

**Authors:** Roberta Bovenzi, Claudio Liguori, Margherita Canesi, Marco D’Amelio, Maria Francesca De Pandis, Carmine Marini, Alessandra Monge, Alessandro Padovani, Alessandro Tessitore, Alessandro Stefani, Mario Zappia, for the SYNAPSES Study Investigators Italian Group, G. Abbruzzese, M. Aguggia, T. Avarello, P. Barone, A. R. Bentivoglio, D. Bosco, P. Calabresi, C. Callegarini, A. Cannas, D. Centonze, R. Ceravolo, C. Colosimo, C. Comi, S. Contardi, P. Cortelli, G. Cossu, V. Di Lazzaro, R. Eleopra, G. Fabbrini, E. Gasparoli, M. Guidi, G. Iliceto, L. Lopiano, P. Manganotti, R. Marconi, M. Mauri, M. Moleri, F. Morgante, A. Negrotti, G. Nordera, M. Onofrj, C. Pacchetti, F. E. Pontieri, A. Priori, L. Provinciali, R. Quatrale, M. Sensi, F. Spagnolo, F. Tamma, M. Tinazzi, C. Vitale, M. A. Volontè, S. Zambito Marsala

**Affiliations:** 1 Department of Systems Medicine, University of Rome Tor Vergata, Via Montpellier 1, 00133 Rome, Italy; 2 Parkinson’s Disease Unit, University Hospital of Rome Tor Vergata, Rome, Italy; 3 Parkinson Institute, ASST Gaetano Pini CTO, Milan, Italy; 4 U.O.C of Neurorehabilitation, Parkinson’s Disease and Movement Disorders Center, Moriggia Pelascini Hospital, Gravedona ed Uniti, Como, Italy; 5 Dipartimento Di Biomedicina, Neuroscienze e Diagnostica Avanzata, Università Degli Studi di Palermo, Palermo, Italy; 6 Department of Human Sciences and Promotion of Quality of Life, San Raffaele University, Rome, Italy; 7 San Raffaele Cassino, Cassino, Italy; 8 Department of Biotechnological and Applied Clinical Sciences, University of L’Aquila, L’Aquila, Italy; 9 Ospedale San Giovanni Battista, ACISMOM, Roma, Italy; 10 Neurology Unit, Department of Clinical and Experimental Sciences, University of Brescia, Brescia, Italy; 11 Department of Advanced Medical and Surgery Sciences, University of Campania “Luigi Vanvitelli”, Naples, Italy; 12 Department “G.F. Ingrassia”, Section of Neurosciences, University of Catania, Catania, Italy

**Keywords:** Parkinson’s disease, Safinamide, MAO-B inhibitor, Safety, Real-life evaluation

## Abstract

**Introduction:**

Safinamide is a recent antiparkinsonian drug that modulates both dopaminergic and glutamatergic systems with positive effects on motor and nonmotor symptoms of Parkinson’s disease (PD). Here, we aimed to describe the efficacy and safety of safinamide in the Italian PD patients in real-life conditions.

**Methods:**

We performed a sub-analysis of the Italian cohort of the SYNAPSES study, a multi-country, multi-center, retrospective-prospective cohort observational study, designed to investigate the use of safinamide in routine clinical practice. Patients received for the first time a treatment with safinamide and were followed up for 12 months. The analysis was conducted on the overall population and in subgroups of interest: i) patients > 75 years, ii) patients with relevant comorbidities and iii) patients affected by psychiatric symptoms.

**Results:**

Italy enrolled 616/1610 patients in 52 centers, accounting for 38% of the entire SYNAPSES cohort. Of the patients enrolled, 86.0% were evaluable at 12 months, with 23.3% being > 75 years, 42.4% with psychiatric conditions and 67.7% with relevant comorbidities.

Safinamide was effective on motor symptoms and fluctuations as measured through the Unified PD rating scale III and IV scores, and on the total score, without safety issues in none of the subgroups considered.

**Conclusion:**

The SYNAPSES data related to Italian patients confirms the good safety profile of safinamide even in special groups of patients. Motor fluctuations and motor impairment improved at the follow-up suggesting the significant role of safinamide in managing motor symptoms in PD patients.

**Supplementary Information:**

The online version contains supplementary material available at 10.1007/s10072-023-07001-6.

## Introduction

Parkinson’s disease (PD) is the second most common neurodegenerative disorder after Alzheimer’s disease [1], characterized by loss of dopaminergic neurons of Substantia Nigra pars compacta (SNpc) and widespread accumulation of α-synuclein containing Lewy Bodies (LBs). Age is the main risk factor for this disease, with prevalence and incidence peaking after 80 years of age [1]. The loss of dopaminergic nigral neurons leads to the cardinal motor symptoms bradykinesia, tremor, and rigidity [1, 2]. Along with motor symptoms, non-motor symptoms, such as pain, fatigue, sleep disorders, gastrointestinal disturbances, and olfactory dysfunction, are often associated with and significantly impact the quality of life (QoL) [1]. Moreover, these non-motor symptoms present more commonly in motor fluctuating PD patients, also influencing the global health status of patients [3].

Therapeutic options in PD remain symptomatic, aiming at controlling motor and non-motor symptoms, and are mostly based on dopaminergic agents [4]; among them, levodopa (L-DOPA) is the gold standard. However, L-DOPA is limited by onset of motor complications with the disease progression [2, 5]. In addition, many motor and non-motor symptoms are resistant to L-DOPA or other dopaminergic drugs [2].

In the neurodegenerative process underlying PD, neurotransmitters other than dopamine are involved, such as noradrenaline, serotonin, acetylcholine, adenosine, and glutamate [2]. Overactive glutamatergic transmission is associated with motor complications (L-DOPA induced dyskinesia, LID), motor symptoms, such as bradykinesia and rigidity, and non-motor symptoms, such as pain, depression, anhedonia, fatigue, urinary and sleep disturbances [6].

Safinamide is the first PD medication having a double mechanism of action, being a reversible monoamine oxidase-B inhibitor (MAOB-i) and a glutamate release modulator through use-dependent sodium and N-type calcium channel blockade [7–10], which differentiate this therapeutic option from the other available MAOB-I on the market, selegiline and rasagiline. In addition to its dopaminergic action, the inhibition of glutamate transmission, with subsequent attenuation of associated excitotoxicity and containment of oxidative stress, might account for a milder profile of neurodegeneration in PD patients treated with safinamide [6]. Due to its multimodal mechanism of action, safinamide is a useful treatment option for PD patients suffering from motor fluctuations and disabling non-motor symptoms [11, 12].

The “SYNAPSES” study (European multicenter retrospective-prospective StudY to observe safiNAmide safety profile and pattern of use in clinical Practice during the firSt post-commErcialization phaSe) was a multinational, multicenter retrospective-prospective cohort observational study, designed, following EMA recommendation, to collect real-life data on safinamide prescription and use in the general population and special groups, not well described in pivotal trials, such as patient older than 75 years, those with relevant comorbidities and those with psychiatric conditions.

The trial was conducted in six European countries (Belgium, Germany, Italy, Spain, Switzerland, and the United Kingdom), involving 1610 patients, of which 25.1% were aged > 75 years, 70.8% had relevant comorbidities and 42.4% had concomitant psychiatric conditions. Patients were followed-up for 12 months. Throughout the study, 45.8% of patients experienced adverse events, mostly mild or moderate, and 27.7% experienced adverse drug reactions, with no differences among groups of interest. A clinically significant improvement was appreciated in the Unified PD rating scale (UPDRS) motor score and the UPDRS total score in more than 40% of patients of the entire population. Significant improvement in motor complications was maintained in the long term [13].

Italy was the first recruiter country, with a total of 616 patients enrolled from 52 specialized centers, accounting for 38% of the entire cohort. Here, we performed a post-hoc analysis of the SYNAPSES trial database to describe the efficacy and safety of safinamide use in the Italian PD population.

## Methods

### Study design and population

We performed a sub-analysis of the Italian cohort of the SYNAPSES study, a multi-country, multi-center, retrospective-prospective cohort observational study, designed to investigate the use of safinamide in routine clinical practice. The study design, inclusion and exclusion criteria, data source, and management have already been described in the original publication by Abruzzese et al. [13].

The study enrolled all patients aged > 18 who received, for the first time, treatment with safinamide at enrollment visit or in the preceding four months. Patients were enrolled for 24 months and were followed up for 12 months, with intermediate evaluations after 4 (± 1), 8 (± 1), and 12 (± 1) months from the start of treatment with safinamide.

A post hoc analysis was performed in the entire Italian cohort and in sub-groups of interest, namely patients aged > 75 years, patients with relevant comorbidities, and patients with psychiatric conditions. The concomitant relevant comorbidities, including psychiatric ones, were those the study investigators considered clinically significant according to their clinical judgment.

Patients were evaluated independently with the UPDRS during ON time. The changes in UPDRS total and motor scores from enrollment to each follow-up visit (4–8-12 months) were computed for each patient.

According to Shulman et al., a clinically important difference was considered in decreases > 4.3 points in the UPDRS total score and > 2.5 points in the UPDRS motor score [14].

The following endpoints were evaluated: main demographic and clinical features, safinamide treatment patterns, concomitant PD and antidepressant medications, efficacy on motor features, non-motor features and motor fluctuations, safety, and tolerability. The proportion of patients experiencing adverse events (AE) and adverse drug reactions (ADR), serious or not, were provided. Both the severity and the potential relationship to the drug were considered according to the investigator’s judgment. The action taken and outcomes have also been reported.

The protocol was approved by all the National and local Independent Ethics Committees and was conducted following the principles of the Helsinki Declaration. All patients signed informed consent. Physicians participating in the study received appropriate compensation.

### Statistical analysis

The statistical analysis was performed on all the Italian patients evaluable for the “Full analysis Set” (FAS), defined as the patients satisfying all inclusion and exclusion criteria.

Patients with missing values were not excluded from the analysis, but their data were not replaced. In case of missing study completion, the observation period end date was computed as the maximum between the following visit date at 4, 8 months follow up and the date of safinamide discontinuation (when applicable).

Descriptive analyses were performed to provide all study endpoints. Categorical variables were described using absolute and relative frequencies; continuous variables using mean, minimum, maximum, standard deviation, and quartiles. Analyses were performed in the overall cohort and in the subgroups of interest: patients > 75 years, patients with relevant comorbidities, and patients with psychiatric conditions.

SAS for Windows Version 9.4 and SAS Enterprise Guide 7.1 was used for statistical analyses.

For a detailed description of statistical methods, see Abbruzzese et al. 2021 [13].

## Results

### Descriptive data

#### Overall cohort features

Fifty-two Italian centers enrolled 616 PD patients, 589 (95.61%) of whom were evaluable for the analysis, and 530 (86%) completed the follow-up at 12 months. Table 1 shows the main features of the global Italian cohort. A comparison of patients’ characteristics with the global population enrolled in the global SYNAPSES study shows no differences between European and Italian PD patients in real-life settings [13]. Most Italian patients were on Hoehn & Yahr stage 2, with a medium latency from disease onset to PD diagnosis of about eight years. Virtually all of patients had motor symptoms (99.8%, in 0.2% this information was missing) and most had non-motor symptoms (87.8%). Among motor features, the most frequently reported symptom was bradykinesia (88.8%), followed by rigidity (84.6%), tremor (53%), and postural instability (27%). Among non-motor symptoms, 46,7% had sleep disorders, 42.4% psychiatric symptoms, 25.6% gastrointestinal symptoms, 23.4% urinary symptoms, 20.9% fatigue, 15.6% pain and 12.8% cognitive disturbances. As expected, almost the entire cohort had motor fluctuations at enrollment (*n* = 571, 96.6%), while the remaining 3.4% had non-motor fluctuations. Regarding motor fluctuations, the observed phenomena included wearing off (84.9%), levodopa-induced-dyskinesias (39.2%), early morning OFF (16.1%), unpredictable OFF (16.1%), delayed-on phenomena (10.2%), or others (8.1%).

**Table 1:** Demographics and main clinical features of the study population

	Total evaluable patients (*N* = 589)	Patients aged > 75 years (*N* = 137)	Patients with relevant comorbidities (*N* = 399)	Patients with psychiatric conditions (*N* = 250)
Sex (male, N, %)	373 (63,3)	86 (62,8)	249 (62,4)	146 (58,4)
Age at enrolment (years) Mean (SD)	68 (9,2)	79,3 (3,0)	69,5 (8,3)	68 (8,7)
Race caucasian (N, %)	588 (99,8)	137 (100)	399 (100)	250 (100)
Diagnosis of Idiopathic PD (N, %)	588 (99,8)	137 (100)	398 (99,7)	249 (99,6)
Time from PD diagnosis (years): mean (SD)	7,7 (5,0)	8,3 (5,3)	7,6 (4,8)	8,6 (5,1)
Time from PD onset of symptoms (years): mean (SD)	8,8 (5,1)	9,3 (5,5)	8,7 (4,9)	9,6 (5,2)
Age at onset of symptoms (years): mean (SD)	59,2 (10,2)	70 (6,6)	60,8 (9,5)	58,3 (9,6)
Hoehn & Yahr stage (N, %)				
1	39 (7,0)	1 (0,8)	19 (5,0)	9 (3,7)
2	312 (55,7)	59 (45,7)	212 (55,8)	111 (46,1)
3	165 (29,5)	49 (38,0)	117 (30,8)	88 (36,5)
4	41 (7,3)	18 (14,0)	30 (7,9)	31 (12,9)
5	3 (0,5)	2 (1,6)	2 (0,5)	2 (0,8)
Motor symptoms: (N, %)	588 (99,8)	137 (100)	399 (100)	249 (99,6)
Non-motor symptoms: (N, %)	517 (87,8)	123 (89,8)	366 (91,7)	249 (99,6)

*N* Number of patients; *SD* Standard deviation

#### Subgroups features

23.3% (*n* = 137) of patients were > 75 years old, 67.7% (*n* = 399) had at least one clinically relevant comorbidity, and 42.4% (*n* = 250) had comorbid psychiatric conditions.

The main comorbidities were blood hypertension and heart diseases (42.8%), and metabolic diseases (20.5%). The main psychiatric symptoms were depression (27.5%) and anxiety (14.1%).

When analyzing the main clinical features of the three subgroups (elderly patients, patients with comorbidities, and with psychiatric conditions), some differences emerged.

As for motor features, tremor and postural instability were more frequent in elderly patients compared to the entire cohort (64.2% vs. 53% and 38% vs. 27%, respectively). Postural instability was more frequent in patients with psychiatric conditions as well (35.2% vs. 27%), along with rigidity (87.2% vs. 84.6%). Among non-motor symptoms, elderly patients presented more frequently urinary symptoms (27.7%), gastrointestinal symptoms (30.7%) and cognitive impairment (23.4%), and slightly higher frequencies of fatigue and pain as well. Patients with psychiatric symptoms had more frequently sleep disturbancies (50%), cognitive impairment (23.4%), and pain (%). Patients with relevant comorbidities did not differ from the overall population in main motor features; however, they showed higher frequencies of some non-motor symptoms such as urinary (27.6%), psychiatric (46.1%), gastrointestinal disturbances (28.6%), and pain (17.5%).

#### Concomitant medications

As for concomitant medications, 100% of patients had at least one at enrollment, mostly levodopa (99.5%), followed by dopamine-agonists (62.6%, mainly pramipexole), COMT inhibitors (20.7%, mainly levodopa/carbidopa/entacapone), amantadine (8.3%), and anticholinergics. (2.2%). Four patients (0.7%) had bilateral STN-DBS. As shown in Supplemental Table 1 (concomitant psychiatric medications at enrollment), about one-quarter of patients (*n* = 148, 25.1%) were receiving antidepressant medications at baseline, mainly SSRI (12.6%), SNRI (5.6%), tricyclics (2.2%), or others (7.3%). Table 2 shows previously discontinued PD treatments by patient age (patients aged < 75 years vs. patients aged > 75 years).

**Table 2:** Discontinued PD treatments by patient age. Percentages are computed out of the total number of Italy evaluable patients for the FAS by patient age

Categories	Active	Patients aged < 75 yrs (*N* = 452)	Patients aged > 75 yrs (*N* = 137)	FAS (*N* = 589)
At least one	Any	81 (17.9%)	17 (12.4%)	98 (16.6%)
COMT inhibitors	Any	5 (1.1%)	0.0	5 (0.8%)
	Carbidopa—Entacapone—Levodopa	5 (1.1%)	0.0	5 (0.8%)
DA	Any	1 (0.2%)	0.0	1 (0.2%)
	Pramipexole dihydrochloride	1 (0.2%)	0.0	1 (0.2%)
Levodopa	Any	11 (2.4%)	1 (0.7%)	12 (2.0%)
	Benserazide hydrochloride—Levodopa	3 (0.7%)	1 (0.7%)	4 (0.7%)
	Carbidopa—Levodopa	2 (0.4%)	0.0	2 (0.3%)
	Carbidopa—Melevodopa	2 (0.4%)	0.0	2 (0.3%)
	Carbidopa—Entacapone—Levodopa	5 (1.1%)	0.0	5 (0.8%)
MAO inhibitors	Any	73 (16.2%)	16 (11.7%)	89 (15.1%)
	Rasagiline	3 (0.7%)	1 (0.7%)	4 (0.7%)
	Rasagiline mesylate	46 (10.2%)	12 (8.8%)	58 (9.8%)
	Rasagiline tartrate	1 (0.2%)	0.0	1 (0.2%)
	Selegiline hydrochloride	23 (5.1%)	3 (2.2%)	26 (4.4%)

A patient could have more than one previous and terminated PD treatments. Carbidopa-Entacapone-Levodopa is shown both as Levodopa and as COMT inhibitors. *COMT* Catechol-O-methyltransferase; *DA* Dopamine agonists; *MAO* monoamine oxidase; *FAS* Full analysis Set

### Safinamide treatment patterns

Safinamide was correctly administered at a 50 mg/day starting dose to 95.9% of the total population; only 21 patients (3.6%) began treatment with 100 mg/day. By the end of the study, 46.7% of patients were receiving safinamide 100 mg/day. Of all Italian patients, 13.2% permanently discontinued safinamide, a lower proportion than those that emerged in the SYNAPSES study (21.6%) and pivotal trials. The proportion of patients discontinuing the drug was lower in those treated with a dose of 100 mg/day (5.4%) than in those treated with 50 mg/day (19.5%). Adverse reactions were the main reason for interruption (*n* = 39, 50%), mainly in patients treated with 50 mg/day (*n* = 35) than in those treated with 100 mg/day (*n* = 4). Other reasons were patients’ choice (*n* = 16, 20.5%) and disease progression (*n* = 5, 6.4%). When analyzing subgroups of interest, patients with psychiatric conditions presented higher rates of drug discontinuation (16.8%) compared to the elderly (10.2%), and those with relevant comorbidities (11.8%).

### Efficacy

#### Motor fluctuations

**Fig. 1:**
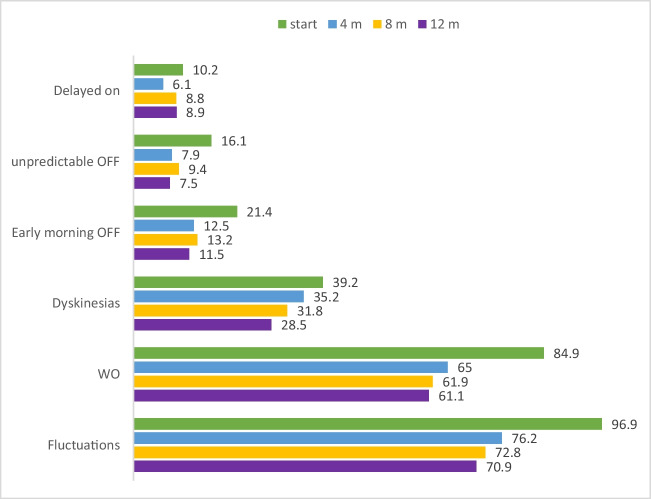
Fluctuations during observation in the overall population (baseline, 4, 8, 12 months). *WO*, wearing-OFF; *m*, month; *start*, baseline

The number of overall patients with motor complications was significantly reduced in the first four months of treatment (*n* = 571, 96.9% at enrollment vs. *n* = 414, 76.2% at four months follow-up) and progressively reduced until the end of the study (*n* = 376, 71% at 12 months follow-up). Among motor fluctuations, the most significative reduction throughout the entire course of the study was observed in unpredictable OFF (-54%), followed by early morning OFF (-28%), wearing OFF (-28%), LIDs (-27%), and delayed ON (-13%) (Fig. 1).

#### UPDRS scores

**Fig. 2:**
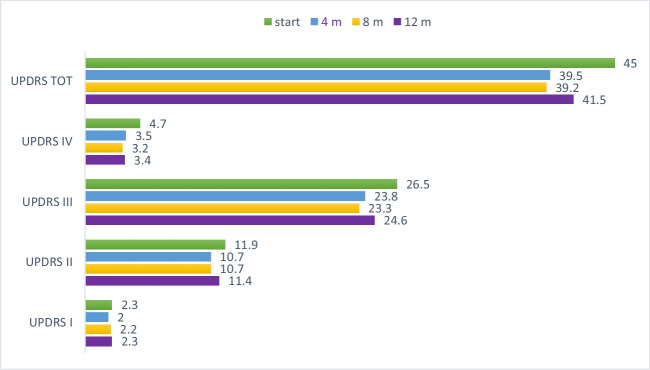
UPDRS I-IV score changes during the trial (baseline, 4, 8, 12 months). *m*, month

Mean UPDRS total and subscales II (activities of daily living), III (motor examination), and IV (motor complications) scores were all improved by safinamide at one-year follow-up (Fig. 2). According to the criteria by Shulman et al., [14] of the 363 evaluable patients at 12 months follow-up, *n* = 137 patients (39%) had a clinically significant improvement in the UPDRS Total score and *n* = 156 (44%) in the UPDRS III scores (Table 3). UPDRS part IV scores improved at 12 months follow-up (4.7 vs. 3.4). When analyzing the three subgroups of patients target of the study SYNAPSES, the proportion of patients with clinically significant improvement in the UPDRS Total score was higher in the elderly than the overall population (43% in patients aged > 75 years vs. 38% in patients aged < 75 years). In contrast, no differences emerged between patients with or without relevant comorbidities or psychiatric conditions. Likewise, the proportion of patients with clinically significant improvement in the UPDRS motor scores was higher in those aged > 75 years (47%) vs those aged < 75 years (43%). Again, no differences emerged between those patients with or without relevant comorbidities, including psychiatric ones. Sub-analyzing subscale II (activities of daily living), an effect of safinamide treatment on tremor and freezing of gait (FOG) emerged, since the first four months of treatment (sub-score 2.10, 35.8% of patients without tremor at enrollment vs. 42.4% at 4 months follow up and 47.3% at 12 months follow up; sub-score 2.13, 11.7% of patients with moderate-to-severe FOG at enrollment vs. 5.1% at 4 months follow up and 6.4% at 12 months follow up).

**Table 3:** Change in UPDRS Total and Motor score after 12 months of treatment with safinamide

	12-months FUP vs baseline (*N* = 363)	
UPDRS: Change in total scores (subscales I, II, and III)	< = −4.3 points	137 (37.7%)
	> −4.3 points*	210 (57.9%)
	NA	16 (4.4%)
UPDRS Part III: Change in motor examination score	< = −2.5 points	156 (43.0%)
	> −2.5 points*	197 (54.3%)
	NA	10 (2.8%)

*FUP* Follow-up; *NA* Not available; *An increase in UPDRS Total score > 4.3 points and in UPDRS Part III > 2.5 points was considered as clinically significant according to Shulman et al. [14]. *UPDRS, Unified Parkinson’s Disease Rating Scale*

UPDRS subscale I (non-motor experiences of daily living) scores were unaffected by safinamide treatment. However, when analyzing sub-score 1.3 (depressed mood), the percentage of patients without depressive symptoms increased after one year of safinamide treatment (39.7% at enrollment vs. 46.5% at 12-month follow-up). Treatment with safinamide reduced the occurrence of sleep disturbances (sub-score 1.7, 47% of patients with sleep problems at enrollment vs 35.7% after 12 months of treatment) and pain as well (sub-score 1.9, 4.8% of patients with frequent pain sensations at enrollment vs 2.3% after 12 months of treatment).

#### Safety and tolerability

Adverse events (AE) were experienced by 27.2% of patients (*n* = 160), and adverse drug reactions (ADR) by 17.1% of them (*n* = 101). However, only 5.4% of patients (*n* = 32) had serious adverse events (SAE), and only 1.2% (*n* = 7) had serious adverse drug reactions (SADR) (Supplemental Fig. 1).

These percentages are lower compared to those observed at a global level in the SYNAPSES study (45.7%) [13] and to those observed in pivotal trials (67%, 67.9%, and 69.5% respectively) [15–17]. Most AE and ADR occurred in patients treated with 50 mg/day (*n* = 328) than those treated with 100 mg/day (*n* = 261). Overall, AEs were mostly mild (70.2%) or moderate (22.2%): 2,8% of them were considered “definitely” related to safinamide. In 60.1% of cases, no specific action was taken; in 35.4%, treatment was permanently or temporarily interrupted; in 4% of them, the dosage of safinamide was reduced from 100 mg/day to 50 mg/day. Of all AE, 60.5% was resolved with or without sequelae, while 11.3% was resolving at the study completion date.

Dyskinesia was the most frequent AE event among nervous system disorders, experienced by 8.5% of the participants (53 events in 50 patients), even if in lower percentages compared to the SYNASPES study (13.7%) [13] and pivotal trials (rates of dyskinesia ranging from 11.9% to 18%) [15–17].

Other AE were psychiatric complications (15.7%, mostly psychomotor agitation), gastrointestinal disturbances (8.1%, mostly nausea), injury (6.9%, mostly femoral fracture), muscle-skeletal disorders (6%, mostly muscular rigidity), and general disorders (6%, mostly edema, and pain).

SAE accounted for 14.9% of all AE (*n* = 37). The main SAE were injuries, including bone fractures (*n* = 8, 3.2%); infections (*n* = 7, 2.8%), and central nervous system disorders, including epilepsy and stroke (*n* = 4, 1.6%). In four cases (1.6%), they caused the participant's death; however, none of them was directly correlated to safinamide treatment. In 28 cases (11.3%), SAE caused a prolongation or a new hospitalization, and in five cases (2%) they caused another major medical event. No significative differences emerged in the three groups of patients of the SYNAPSES study (patients aged > 75 years, those with relevant comorbidities, and those with psychiatric conditions) (Table 4). Patients with psychiatric conditions had higher rates of AEs (30%) than patients without psychiatric comorbidities (about 25%); however, this difference did not reach statistical significance. Supplemental Fig. 2 shows the main differences in safety outcomes between groups.

**Table 4:** Impact on adverse events occurrence of demographic and clinical factors

		Patients analysed (*N* = 589)	Patients with AE (*N* = 160)	Patients without AE (*N* = 429)	Test	*p *value
Patients age	Patients aged < = 75 yrs	452 (76.7%) °	124 (27.4%) °°	328 (72.6%) °°	Chi-square test	0.7898
	Patients aged > 75 yrs	137 (23.3%)	36 (26.3%)	101 (73.7%)		
Relevant comorbidities	Relevant comorbidities	399 (67.7%)	108 (27.1%)	291 (72.9%)	Chi-square test	0.9389
	No relevant comorbidities	190 (32.3%)	52 (27.4%)	138 (72.6%)		
Psychiatric conditions	Psychiatric conditions	250 (42.4%)	75 (30%)	175 (70%)	Chi-square test	0.1840
	No psychiatric conditions	339 (57.6%)	85 (25.1%)	254 (74.9%)		
Ongoing treatment at safinamide start	Levodopa	176 (30.0%)	43 (24.4%)	133 (75.6%)	Fisher exact test	0.7030
	Levodopa + 1 treatment	286 (48.8%)	80 (28%)	206 (72%)		
	Levodopa + > = 2 treatments	124 (21.2%)	34 (27.4%)	90 (72.6%)		
	UNK	3	3	0		

° Percentages computed over the number of Italian evaluable patients with non-missing values (*FAS*, Full Analysis Set); °° Percentages computed over the number of patients of each subgroup of interest. *UNK* refers to three individuals who were receiving only dopamine agonists at the time of safinamide administration. *AE*, adverse events

## Discussion

In this study, we conducted a sub-analysis of the SYNAPSES trial in a representative Italian population of PD patients. Our findings confirmed the overall safety, tolerability, and efficacy of safinamide in real-life settings across the Italian country, even in frailer groups of patients, such as those aged > 75 years, patients with relevant comorbidities, and those with psychiatric conditions. Most importantly, safinamide was well tolerated, with no major or unexpected safety concerns. The overall rates of AEs in the Italian population were almost 20% lower than those found in the global SYNAPSES study and about 40% lower than pivotal studies [13, 15, 16]. This is noteworthy because pivotal trials enrolled patients generally not affected by comorbidities and usually more selected than subjects included in real-world studies. In this sub-analysis, AEs were predominantly mild to moderate in intensity, and the most serious ones were not related to the investigational drug. As in the European population, the main AE observed in the Italian cohort was dyskinesia (8.5%), which, in trend with other AEs, occurred at lower rates compared to the European population (13.7%) [13] and pivotal trials (18.3% and 14.6%, respectively) [15, 16]. Dyskinesia was mostly reported since the beginning of the study and was not further aggravated by the safinamide treatment. However, dyskinesia in safinamide studies present usually a two sides coin in the clinician’s hand: it can emerge and be considered an AE, but it can be reduced due to the beneficial effect of the drug on motor symptoms. Accordingly, safinamide reduced the occurrence of dyskinesia in 27% of patients. The antidyskinetic effect of safinamide was already described, and comes from preclinical [18] and clinical studies, as demonstrated in post-hoc dedicated analyses of the 016/018 trial [19, 20]. The antidyskinetic activity of safinamide may be related to its dual mechanism of action. On the one hand, its dopaminergic activity might allow better motor control with no need to change or increase the concomitant dopaminergic therapy. Indeed, in a recent publication, Cilia et al. estimated that safinamide 50 corresponds to 100 mg of levodopa whereas safinamide 100 mg to 125 mg of levodopa, demonstrating that high doses of safinamide allow lower doses of levodopa-based medications and overall simplification of the therapeutic scheme [21], potentially reducing the risk of some dopaminergic adverse effects, and improving patient adherence to treatment. On the other hand, the glutamatergic action of safinamide 100 mg might reduce the excitatory overdrive of the direct pathway and the abnormal cortical facilitation both implied in levodopa-induced-dyskinesias pathophysiology. The alteration in motor circuits in PD can be normalized with safinamide 100 mg/day, and this effect persists after long-term treatment suggesting a modulation of synaptic plasticity [22].

The good safety profile of safinamide was further supported by the absence of serotoninergic syndromes despite about one-quarter of patients were contextually receiving antidepressant medications. Unlike other MAO-B inhibitors selegiline and rasagiline, the inhibition of safinamide is reversible, and highly selective, thus minimizing the risk of hypertensive crises or serotonergic syndrome [23]. Furthermore, safinamide is not metabolized by cytochrome P-450 and this avoids major pharmokinetic interactions with other drugs, making safinamide a safer option for polytherapy-treated PD patients.

Considering non-motor symptoms associated with dopaminergic and glutamatergic networks, safinamide introduction did not aggravate impulse control disorders or sleep disturbances, both influenced by dopamine and glutamate levels [24, 25] and potentially worsened by specific dopaminergic agents [26, 27]. This result concords with previous literature suggesting a beneficial effect of safinamide on sleep in PD patients [11, 12]. Considering the impact of sleep disturbances on well-being of PD patients, the improvement in non-motor symptoms demonstrated by safinamide was also associated with the increased patients’ quality of life [11, 28]. This sub-analysis also confirmed a positive effect of safinamide on depression, fatigue, and urinary symptoms.

Finally, no significative differences in AEs were found in patients over 75 years, with comorbidities and psychiatric conditions, confirming that safinamide does not require special safety precautions in these groups of subjects.

We have also found several beneficial effects of safinamide in all the groups of patients. Motor fluctuations affected virtually the entire cohort of patients in this post-hoc analysis, considering the indication of the drug. The pharmacological approach to motor fluctuations is a significant challenge as it carries the risk of inducing or exacerbating dyskinesias [15]. In pivotal studies, safinamide has been shown to significantly reduce OFF time up to > 1 h per day compared to placebo and increase ON time without troublesome dyskinesias [15, 29]. A growing body of evidence supports the efficacy of switching from classical MAO-B inhibitors to high-dose safinamide in the treatment of residual wearing-OFF [30–32]. Our sub-analysis confirmed the beneficial effect of safinamide on motor fluctuations, mostly unpredictable OFF (-54% of patients affected) and morning OFF phenomena (-47%). The beneficial effect was evident since the first three months of treatment and was sustained throughout time [13].

Motor impairment globally improved under safinamide treatment, since about 44% of patients presented a clinically significant improvement in UPDRS motor scores after one year of safinamide treatment. These results align with the findings of the global SYNAPSES trial, where 45% of the entire cohort reached the same improvement.

When analyzing the UPDRS part II sub-scores, we found an effect of safinamide on tremor and freezing of gait, both characterized by a variable response to levodopa therapy [33]. Data from preclinical [33] and post-hoc registration studies [34, 35] support a possible tremorlytic effect of safinamide that might be driven by the heterogeneous action of the drug. However, further dedicated studies are needed.

Along with motor symptoms, non-motor symptoms represent a major burden for PD patients, especially in fluctuating ones [3]. Compelling evidence supports a beneficial effect of safinamide on non-motor symptoms, such as mood [36, 37], cognition [38, 39], sleep [11, 12], and pain [40], which might be driven the multimodal action of the drug on the dopaminergic and glutamatergic systems.

Among non-motor symptoms, neuropsychiatric manifestations account for the greatest reduction in quality of life [41]. In this study, 28% of patients had some depressive symptoms, according to the investigator’s judgment; of them, about 7% no longer reported mood deflection after one year of safinamide treatment. Sleep disturbances also represent a common and disabling non-motor symptoms in PD [11], affecting almost 50% of patients in our cohort at enrollment. One-year treatment with safinamide reduced the rate of patients suffering with sleep disturbances of about 15%. Finally, in this post-hoc analysis, safinamide treatment halved the quote of PD patients suffering with pain, one of the most underestimated and inadequately treated NMS.

The beneficial effect of safinamide on PD was further supported by a clinically significant reduction in total UPDRS scores in about 40% of patients after one year of safinamide treatment. As for the motor scores, the most profound effect was seen between months four and eight of the follow-up, and long lasted until the end of the study. Interestingly, patients aged > 75 years had greater rates of clinically significant improvement in both total and motor UPDRS scores. In contrast, comorbidities and psychiatric conditions did not influence the clinical response to the drug. These data suggest that safinamide could be not only safe, but remarkably effective in the elderly PD population.

Limitations of this study include the open-label design, which lacked a placebo or active control group, as well as the post-hoc analyses.

Despite its limitations, this sub-analysis of the SYNAPSES study confirms the efficacy and safety of safinamide use in the Italian PD population. The safety profile of safinamide was confirmed in frailer groups of patients, namely those aged > 75 years and patients with relevant comorbidities, including psychiatric ones. Finally, this post-hoc analysis provided crucial information about several motor and non-motor features that did not emerge from the overall trial. These findings suggest that safinamide could be a safe and effective treatment option for a broader range of Parkinson's disease patients, including those with age-related and psychiatric comorbidities. Further research is needed to confirm these results and examine the potential benefits of safinamide for treating specific motor and nonmotor symptoms.

### Supplementary Information

Below is the link to the electronic supplementary material.

Supplementary file1 (DOCX 142 kb)

## Data Availability

Data is available from the corresponding author upon reasonable request.
