# Supplementary material for: Real-world use of Safinamide in motor fluctuating Parkinson’s disease patients in Italy
Source: Neurol Sci. 2023 Sep 9;45(2):573–83. doi: 10.1007/s10072-023-07001-6 (PMC10791801; doi:10.1007/s10072-023-07001-6)
Supplement: Supplementary file 1 — Supplementary file1 (DOCX 142 kb) [file 10072_2023_7001_MOESM1_ESM.docx]

**Supplemental Table 1.** Concomitant psychiatric medications at enrolment (*N,* %). *SNRI,* Serotonin–norepinephrine reuptake inhibitors; *SSRI*, Selective Serotonin reuptake inhibitors.

| **Categories** |  | **Total number of Italy evaluable patients (FAS) (N= 589)** |
| --- | --- | --- |
| Antidepressant | Any | 148 (25.1%) |
|  | SNRI | 33 (5.6%) |
|  | SSRI | 74 (12.6%) |
|  | Tricyclic | 13 (2.2%) |
|  | Other Antidepressant | 43 (7.3%) |
| Antipsychotics | Any | 47 (8.0%) |
| Procholinergics | Any | 13 (2.2%) |
| Other Psychiatric | Any | 140 (23.8%) |

**Supplemental Figure 1.** Adverse events and adverse reactions during observation. *AE*, adverse events; *SAE*, serious adverse events*; ADR*, adverse drug reaction; *SADR*, serious adverse drug reactions.

**Supplemental Figure 2.** Safety summary in subgroups. *AEs*, adverse events. The differences found between groups did not reach statistical significance.
